# Higher systolic blood pressure is specifically associated with better islet beta-cell function in T2DM patients with high glycemic level

**DOI:** 10.1186/s12933-022-01723-1

**Published:** 2022-12-19

**Authors:** Zhang Xia, Lijuan Song, Dongdong Fang, Wenjun You, Feng Li, Deqiang Zheng, Yuhao Li, Lu Lin, Jingtao Dou, Xin Su, Qi Zhai, Yingting Zuo, Yibo Zhang, Herbert Y. Gaisano, Jiajia Jiang, Yan He

**Affiliations:** 1 Department of Epidemiology and Health Statistics, School of Public Health, Capital Medical University, No.10 Xitoutiao, You’anmen Wai, Fengtai District, Beijing, 100069 China; 2 Department of Endocrinology, Jining No.1 People’s Hospital, 6 Jiankang Road, Rencheng District, Jining, Shandong 272000 China; 3 Institute for Chronic Disease Management, Jining No.1 People’s Hospital, Jining, Shandong China; 4 Beijing Municipal Key Laboratory of Clinical Epidemiology, Beijing, China; 5 Department of Endocrinology, The First Medical Center, Chinese PLA General Hospital, Beijing, China; 6 Departments of Medication and Physiology, University of Toronto, Toronto, ON Canada

**Keywords:** Type 2 diabetes mellitus, Beta-cell function, Blood pressure, Mediating effect

## Abstract

**Background:**

Patients with type 2 diabetes mellitus (T2DM) usually have higher blood viscosity attributed to high blood glucose that can decrease blood supply to the pancreas. A mild increase in blood pressure (BP) has been reported as a potential compensatory response that can maintain blood perfusion in the islet. However, how BP influences beta-cell function in T2DM subjects remains inconsistent. This study aimed to examine the relationship between BP and beta-cell function in patients with T2DM under different HbA1c levels.

**Methods:**

This is a cross-sectional study of 615 T2DM patients, whose clinical data were extracted from hospital medical records. Beta-cell function was assessed by insulin secretion-sensitivity index-2 (ISSI2). Multivariable linear regression analysis and restricted cubic splines (RCS) analysis were performed to identify the association between systolic BP (SBP) and ISSI2. Mediation analysis was performed to determine whether higher SBP could reduce blood glucose by enhancing beta-cell function.

**Results:**

After adjustment of potential confounders, in participants with HbA1c ≥ 10%, the SBP between 140 to150 mmHg had the highest log ISSI2 (*b* = 0.227, 95% CI 0.053–0.402), an association specific to participants with < 1 year duration of diabetes. RCS analyses demonstrated an inverted U-shaped association between SBP and ISSI2 with the SBP at 144 mmHg corresponding to the best beta-cell function. This higher SBP was “paradoxically” associated with lower 2 h postprandial blood glucose (PBG) when SBP < 150 mmHg that was almost exclusively mediated by ISSI2 (mediating effect = − 0.043, 95%CI − 0.067 to − 0.018; mediating effect percentage = 94.7%, *P* < 0.01). SBP was however not associated with improvement in ISSI2 or 2 h PBG in participants with HbA1c < 10%.

**Conclusions:**

In early stage of diabetes, a slightly elevated SBP (140–150 mmHg) was transiently associated with better beta-cell function in T2DM patients with HbA1c ≥ 10% but not in those with HbA1c < 10%.

**Supplementary Information:**

The online version contains supplementary material available at 10.1186/s12933-022-01723-1.

## Background

Normal islet beta-cell function is critical for maintaining blood glucose homeostasis. Beta-cell function is vulnerable to changes in islet blood supply, which ensures delivery of nutrients and oxygen to islet cells, removes metabolic wastes and circulates the released hormones [1]. Endocrine cells constitute only ~ 1% of total pancreatic cell mass but consume 5–15% of the blood supply to pancreas [2].

Blood pressure (BP) is the driving force of organ perfusion. Animal models were used to show that elevated BP promoted islet blood supply, resulting in increased number and volume of islet cells and consequent enhancement of glucose-stimulated insulin secretion [3]. Some clinical studies also support the importance of elevated BP in organ perfusion in patients with diabetes. In patients with type 2 diabetes mellitus (T2DM), the increased blood viscosity induced by hyperglycemia and some erythrocyte-related abnormalities together can reduce the blood flow rate, which would require raising the BP as a compensatory mechanism to avoid tissue ischemia [4–7]. A positive association between blood viscosity and a higher BP was observed in both diabetic and non-diabetic individuals [8, 9]. This led us to hypothesize that a relatively higher BP might enhance beta-cell function in T2DM patients that have a high glycemic level.

The population-based evidence on the relationship between BP and beta-cell function has been inconsistent. Some studies from newly diagnosed T2DM patients and non-diabetic individuals demonstrated that higher systolic BP (SBP) was associated with improved beta-cell function [10, 11]. In contrast, other studies found that hypertension or elevated SBP was a risk factor for beta-cell dysfunction [12–14]. Notably, there are no study that examines what range of SBP corresponds to better islet beta-cell function and whether this association may be different in T2DM patients with different glycemic status.

A better beta-cell function is associated with not only favorable metabolic control but also decreased risk of cardiovascular complications and hypoglycemia [15–17]. Currently, a paradigm shift from the glucose-centric to beta cell-centric concept has been proposed in the management of T2DM, which emphasizes the importance of beta-cell preservation rather than just lowering the glycosylated hemoglobin (HbA1c) level [18]. Given the approximately 80% rate of hypertension prevalence in adults with diabetes and nearly 50% rate of inadequate glycemic control in treated T2DM patients [19, 20], there is urgent need to clarify the relationship between BP and islet beta-cell function in order to optimize the latter.

In this study, we performed the analysis in T2DM patients with different HbA1c levels to examine the relationship between BP and beta-cell function, with the aim to determine the optimal BP that ensures better beta-cell function which we hypothesize is different at the different glycemic levels.

## Methods

### Study design and participants

This was a cross-sectional study. We enrolled 623 adult (age ≥ 18 years) patients with T2DM who were in hospital due to poor glycemic control from January 2019 to July 2022 at the Department of Endocrinology, Jining First People’s Hospital that is affiliated to the Jining Medical University, Jining, China. Patients with acute pancreatitis, pancreatic atrophy, and SBP < 90 mmHg or diastolic BP (DBP) < 60 mmHg were excluded. Finally, a total of 615 patients were included in the analysis. The study protocol was approved by the Medical Ethics Committee of the Jining Medical University (No. JNMC-2022-YX-019).

### Clinical and laboratory measurements

All data were extracted from hospital medical records by trained staff using the quality-control Epidata version 3.1 software.

Demographics, lifestyle, history of chronic diseases, duration of diabetes, medications used were collected. Body height and weight were measured by height and weight meters (OMRON HNH-318; OMRON Corporation, Shenzhen, China). Blood pressure was measured by an electronic BP meter (OMRON HBP-1100U; OMRON Corporation, Dalian, China) in the seated position, with feet on the floor and arm supported at heart level. Laboratory examination items, including lipid profiles and hepatorenal function, were tested enzymatically by an automatic biochemistry analyzer (AU5831, Beckman Coulter, USA). HbA1c was tested by a hemoglobin analyzer (Bio-Rad D-10) using high-pressure liquid chromatography. All information above was collected at the time of admission of the patients.

A standardized steamed bread meal test (SBMT) was conducted before patient discharge. The night before the SBMT, patients stopped taking medications that would affect the trial and kept overnight fasting. The next morning, before patients took medications, venous blood samples were drawn to measure blood glucose, insulin, and C-peptide at fasting, 60 min, 120 min, and 180 min following the ingestion of 100-g flour. Insulin and C-peptide were tested by an automatic electrochemiluminescence analyzer (Cobas e801, Roche Diagnostics, Mannheim, Germany). Blood glucose was tested enzymatically by an automatic biochemistry analyzer (AU5831, Beckman Coulter, USA).

### Definitions

The diagnosis of T2DM was made according to the 1999 World Health Organization criteria: fasting blood glucose (FBG) ≥ 7.0 mmol/L or 2-h oral glucose tolerance test plasma glucose ≥ 11.1 mmol/L or self-reported physician-diagnosed diabetes. Hypertension was defined as SBP ≥ 140 mmHg and/or DBP ≥ 90 mmHg, self-reported physician-diagnosed hypertension, or taking antihypertensive medications even with SBP < 140 mmHg or DBP < 90 mmHg. Overweight was defined as body mass index (BMI) of ≥ 25 kg/m^2^ but < 30 kg/m^2^, and obesity is BMI ≥ 30 kg/m^2^, according to the American Heart Association recommendations [21].

### Index calculation

BMI was calculated as weight (kg)/[height (m) × height (m)]. Insulin secretion-sensitivity index-2 (ISSI2) = (AUC_insulin_/AUC_glucose_) × Matsuda, where AUC_insulin_ and AUC_glucose_ are the areas under the insulin curve and glucose curve within 120 min of SBMT [22]. Matsuda = 10,000/√(MG × MI) × (FG × FI), where MG is mean glucose, MI is mean insulin, FG is fasting glucose, and FI is fasting insulin. Homeostatic model assessment 2-insulin resistance (HOMA2-IR) and homeostatic model assessment 2-beta (HOMA2-B) were calculated by HOMA2 model based on blood glucose and C-peptide [23]. The unit of blood glucose, insulin, and C-peptide were mmol/L, μIU/ml, and nmol/L, respectively. Antihypertensive treatment rate = (number of participants undergoing oral antihypertensive agent treatment/number of participants with hypertension) × 100%.

### Statistical analysis

We used spline function in the RStudio version 1.4.1106. to estimate the blood glucose, insulin, and C-peptide at 30 min of SBMT. We divided participants into two groups based on the threshold of 10% of HbA1c and performed analyses in each group.

Continuous variables with normal distribution were presented as mean ± SD and compared by two independent sample t-test or one-way analysis of variance. Continuous variables with skewed distribution were presented as median (25th–75th) and compared by Wilcoxon rank sum test or Kruskal–Wallis H test. Categorical variables were presented as numbers (percentages) and compared by Chi-square test.

To identify the association between BP and beta-cell function (ISSI2), multivariable linear regression analysis was performed with adjustment of age, sex, duration of diabetes, BMI, triglyceride (TG), low-density lipoprotein cholesterol (LDL-C), Matsuda index, and medications used. BP was analyzed in the model as a continuous variable and a categorical variable, respectively. Among participants with HbA1c ≥ 10%, we performed sensitivity analyses in following subjects: insulin sensitivity in 10th–90th percentiles, without taking oral antihypertensive agents, without accepting insulin treatment, and duration of diabetes < 1 year or ≥ 1 year.

To identify the dose–response relationship between SBP and ISSI2, we conducted restricted cubic splines (RCS) analyses. We set four knots at the 5th, 25th, 75th, and 95th percentiles, and set SBP of 120 mmHg as a reference. In addition, to determine the dose–response relationship between SBP and blood glucose, we further analyzed the association between SBP and blood glucose by RCS with the same parameters set. *P*_overall_ < 0.05 and *P*_non-linear_ < 0.05 indicated that the association was nonlinear.

To determine whether beta-cell function mediated the association between SBP and 2 h postprandial blood glucose (PBG) in participants with HbA1c ≥ 10%, we performed mediation analyses. Bootstrap method was used to estimate the confidence interval with the seed set to 5000.

All statistical analyses were conducted in SAS version 9.4 (SAS Institute Inc, Cary, NC). A two-tailed *P* value < 0.05 was considered statistically significant.

## Results

### Characteristics of participants

This study included 615 T2DM participants with 411 (66.8%) male and a mean age of 49.2 ± 12.1 years. The mean HbA1c for all participants was 9.8 ± 2.1% (83.6 ± 23.4 mmol/mol) and the median duration of diabetes was 3.7 (0.1–10.0) years. Compared to those with HbA1c < 10%, participants with HbA1c ≥ 10% were younger (46.3 ± 12.3 years vs. 51.5 ± 11.4 years, *P* < 0.01), had a shorter duration of diabetes [1.0 (0.1–7.0) years vs. 5.6 (1.3–10.7) years, *P* < 0.01], a lower prevalence of self-reported hypertension (27.6% vs. 35.6%, *P* < 0.05), coronary heart disease (8.5% vs. 20.3%, *P* < 0.01), stroke (6.4% vs. 12.8%, *P* < 0.01); and manifested a lower level of beta-cell function [97.1 (66.5–136.5) vs. 141.0 (87.9–198.0), *P* < 0.01]. Amongst all the hypertensive subjects, those that were on antihypertensive treatment was 53.1% (172/324), which when divided into those with HbA1c < 10% versus ≥ 10%, it was 57.6% (110/191), and 44.7% (55/123), respectively (Table 1).

**Table 1:** Demographic and clinical characteristics of participants

Characteristics	All participants	Participants with HbA1c < 10%	Participants with HbA1c ≥ 10%	*P* value^*^
N	615	320	283	
Age, mean ± SD, year	49.2 ± 12.1	51.5 ± 11.4	46.3 ± 12.3	< 0.01
Male sex, n (%)	411 (66.8)	206 (64.4)	197 (69.6)	0.17
Current smoking, n (%)	198 (32.4)	95 (29.9)	102 (36.2)	0.10
Current alcohol consumption, n (%)	280 (45.8)	138 (43.4)	139 (49.3)	0.15
Duration of diabetes, median (25th–75th), year	3.7 (0.1–10.0)	5.6 (1.3–10.7)	1.0 (0.1–7.0)	< 0.01
Hypertension, n (%)	324 (52.7)	191 (59.7)	123 (43.5)	< 0.01
Self-reported hypertension, n (%)	198 (32.2)	114 (35.6)	78 (27.6)	0.03
Newly diagnosed hypertension, n (%)	126 (20.5)	77 (24.1)	45 (15.9)	0.01
CHD, n (%)	92 (15.0)	65 (20.3)	24 (8.5)	< 0.01
Stroke, n (%)	62 (10.1)	41 (12.8)	18 (6.4)	< 0.01
BMI, mean ± SD, kg/m^2^	25.9 ± 3.7	26.1 ± 3.9	25.7 ± 3.6	0.31
SBP, mean ± SD, mmHg	131.3 ± 17.2	133.0 ± 17.2	129.2 ± 17.0	< 0.01
DBP, mean ± SD, mmHg	80.9 ± 10.5	81.3 ± 10.5	80.4 ± 10.6	0.29
HbA1c, mean ± SD, %	9.8 ± 2.1	8.2 ± 1.2	11.6 ± 1.3	< 0.01
HbA1c, mean ± SD, mmol/mol	83.6 ± 23.4	65.8 ± 12.7	103.7 ± 14.6	< 0.01
FBG, mean ± SD, mmol/L	8.3 ± 2.3	7.6 ± 2.0	8.9 ± 2.5	< 0.01
2 h PBG, mean ± SD, mmol/L	17.0 ± 4.6	16.0 ± 4.5	18.0 ± 4.5	< 0.01
TG, median (25th–75th), mmol/L	1.5 (1.0–2.5)	1.5 (1.0–2.4)	1.5 (1.0–2.7)	0.68
TC, median (25th–75th), mmol/L	4.5 (3.8–5.2)	4.4 (3.8–5.1)	4.7 (4.0–5.4)	< 0.01
HDL-C, median (25th–75th), mmol/L	1.0 (0.8–1.2)	1.0 (0.8–1.2)	1.0 (0.8–1.2)	0.96
LDL-C, median (25th–75th), mmol/L	2.6 (2.0–3.2)	2.5 (2.0–3.1)	2.8 (2.2–3.4)	< 0.01
ALT, median (25th–75th), U/L	19.1 (13.3–29.4)	19.1 (13.3–29.4)	18.9 (13.4–28.3)	0.82
AST, median (25th–75th), U/L	15.9 (12.7–21.8)	16.3 (13.4–22.0)	15.1 (12.0–20.2)	< 0.01
UA, median (25th–75th), μmol/L	286.0 (232.0–348.0)	295.0 (245.0–356.5)	274.0 (216.0–334.0)	< 0.01
Oral antihypertensive agent treatment (not mutually exclusive below), n (%)	172 (28.0)	110 (34.4)	55 (19.4)	< 0.01
Calcium channel blocker, n (%)	94 (15.3)	57 (17.8)	33 (11.7)	0.03
ACE inhibitor/ARB, n (%)	127 (20.7)	79 (24.7)	46 (16.3)	0.01
Alpha blocker/Beta blocker, n (%)	82 (13.3)	57 (17.8)	21 (7.4)	< 0.01
Diuretic, n (%)	50 (8.1)	28 (8.8)	20 (7.1)	0.45
Insulin treatment, n (%)	140 (22.8)	80 (25.0)	54 (19.1)	0.08
Oral hypoglycemic agent treatment, n (%)	382 (62.1)	236 (73.8)	137 (48.4)	< 0.01
HOMA2-IR, median (25th–75th)	1.7 (1.3–2.3)	1.8 (1.3–2.4)	1.7 (1.4–2.2)	0.11
Matsuda index, median (25th–75th)	81.3 (54.3–120.4)	74.7 (48.4–106.5)	90.2 (63.5–133.7)	< 0.01
HOMA2-B, median (25th–75th)	51.5 (36.3–80.0)	64.7 (41.7–95.7)	44.7 (30.8–61.0)	< 0.01
ISSI2, median (25th–75th)	114.8 (77.0–172.7)	141.0 (87.9–198.0)	97.1 (66.5–136.5)	< 0.01

CHD, coronary heart disease; BMI, body mass index; SBP, systolic blood pressure; DBP, diastolic blood pressure; HbA1c, glycosylated hemoglobin; FBG, fasting blood glucose; PBG, postprandial blood glucose; TG, triglyceride; TC, total cholesterol; HDL-C, high-density lipoprotein cholesterol; LDL-C, low-density lipoprotein cholesterol; ALT, alanine transaminase; AST, aspartate aminotransferase; UA, uric acid; ACE, angiotensin converting enzyme; ARB, angiotensin receptor blocker; HOMA2-IR, homeostatic model assessment 2-insulin resistance; HOMA2-B, homeostatic model assessment 2-beta; ISSI2, insulin secretion-sensitivity index-2

^*^Compared the difference between two HbA1c groups

### Higher SBP was associated with better beta-cell function in participants with HbA1c ≥ 10%

To examine whether a relatively higher BP level might enhance the beta-cell function in T2DM patients with a high glycemic level, we first performed the multivariable linear regression analysis in participants with HbA1c < 10% versus ≥ 10%, respectively. In participants with HbA1c ≥ 10%, the DBP was positively associated with log ISSI2 (*b* = 0.058, 95% CI 0.007–0.110), and SBP was marginally associated with log ISSI2 (*b* = 0.023, 95% CI − 0.011 to 0.056) after adjustment for age, sex, duration of diabetes, BMI, log TG, LDL-C, log Matsuda index, insulin treatment, oral hypoglycemic agent treatment, ACE inhibitor/ARB treatment, and other antihypertensive agent treatment (Table 2).

**Table 2:** Association between blood pressure and ISSI2 in participants with HbA1c < 10% or ≥ 10%

Factor	Participants with HbA1c < 10%			Participants with HbA1c ≥ 10%		
	N	ISSI2	*b* (95%CI)	N	ISSI2	*b* (95%CI)
SBP model						
Continuous variable						
SBP, per 10 mmHg	308	141.3 (89.2–198.7)	− 0.013 (− 0.047 to 0.021)	271	97.6 (66.5–138.9)	0.023 (− 0.011 to 0.056)
Categorical variable						
90 ≤ SBP < 120, mmHg	73	150.4 (95.3–206.9)	Ref.	87	90.7 (67.6–127.9)	Ref.
120 ≤ SBP < 130, mmHg	62	143.5 (85.5–196.2)	− 0.007 (− 0.179 to 0.165)	64	110.6 (75.1–150.9)	0.142 (0.001–0.283)
130 ≤ SBP < 140, mmHg	71	145.5 (108.0–213.1)	0.033 (− 0.134 to 0.200)	54	95.9 (63.6–135.2)	0.121 (− 0.029 to 0.271)
140 ≤ SBP < 150, mmHg	50	140.5 (86.3–185.8)	− 0.125 (− 0.314 to 0.063)	35	108.6 (65.3–188.2)	0.227 (0.053–0.402)
150 ≤ SBP, mmHg	52	109.3 (86.2–179.1)	− 0.030 (− 0.215 to 0.154)	31	91.8 (60.2–132.8)	0.116 (− 0.082 to 0.314)
DBP model						
Continuous variable						
DBP, per 10 mmHg	308	141.3 (89.2–198.7)	− 0.018 (− 0.073 to 0.037)	271	97.6 (66.5–138.9)	0.058 (0.007–0.110)
Categorical variable						
60 ≤ DBP < 80, mmHg	145	140.9 (85.5–197.7)	Ref.	134	90.9 (66.0–128.7)	Ref.
80 ≤ DBP < 90, mmHg	90	135.0 (92.7–195.7)	− 0.024 (− 0.156 to 0.108)	88	97.3 (63.1–142.7)	0.084 (− 0.034 to 0.203)
90 ≤ DBP, mmHg	73	147.3 (100.8–207.8)	− 0.020 (− 0.165 to 0.125)	49	114.8 (81.2–146.2)	0.088 (− 0.060 to 0.235)

Data of ISSI2 were median (25th–75th). *b*(95%CI) was from linear regression analysis. ISSI2 was analyzed in models with the form of log transformation. All models adjusted age, sex, duration of diabetes, BMI, log TG, LDL-C, log Matsuda index, insulin treatment, oral hypoglycemic agent treatment, ACE inhibitor/ARB treatment, and other antihypertensive agent treatment. ISSI2, insulin secretion-sensitivity index-2; SBP, systolic blood pressure; DBP, diastolic blood pressure; HbA1c, glycosylated hemoglobin; BMI, body mass index; TG, triglyceride; LDL-C, low-density lipoprotein cholesterol; ACE, angiotensin converting enzyme; ARB, angiotensin receptor blocker

Then, we divided the participants into different SBP groups (90–120 mmHg group and the other four groups at 10 mmHg increment from 120 to150 mmHg) or DBP groups (60–80 mmHg group and the other two groups at 10 mmHg increment from 80 to 90 mmHg), and then compared their ISSI2. The characteristics of participants with different BP groups in HbA1c < 10% or ≥ 10% were demonstrated in the Additional file 1: Table S1 or Table S2. The level of HbA1c, FBG, 2 h PBG, TG, total cholesterol (TC), high-density lipoprotein cholesterol (HDL-C), and LDL-C were not significantly different among these SBP groups or DBP groups. Remarkably, we found that a SBP between 140–150 mmHg had the highest log ISSI2 (*b* = 0.227, 95% CI 0.053–0.402) in participants with HbA1c ≥ 10%, but this phenomenon was not found in participants with HbA1c < 10%. The DBP was not associated with log ISSI2 either in participants with HbA1c ≥ 10% or < 10% (Table 2).

To evaluate the robustness of these results in participants with HbA1c ≥ 10%, we conducted sensitivity analyses in the following subjects: (1) insulin sensitivity in 10th–90th percentiles; (2) without taking oral antihypertensive agents; (3) without accepting insulin treatment; (4) duration of diabetes < 1 year; (5) duration of diabetes ≥ 1 year. This robust sensitivity analysis employing all these parameters supported the observation that a SBP of 140–150 mmHg was associated with the better log ISSI2 except for the parameter of the duration of diabetes ≥ 1 year. However, the DBP was not associated with log ISSI2 improvement in the same sensitivity analyses (Additional file 1: Table S3), indicating the previous result that DBP as a continuous variable shown to be positively associated with log ISSI2 was not a robust finding. Thus, in the subsequent analyses, we did not further analyze the relationship between DBP and beta-cell function.

**Fig. 1:**
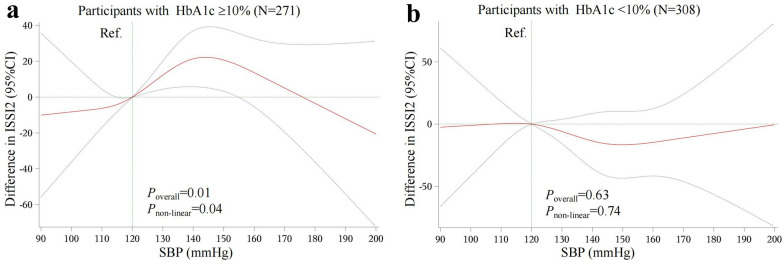
RCS analysis on the association between SBP and ISSI2. **a** association in participants with HbA1c ≥ 10%. **b** association in participants with HbA1c < 10%. *P*_overall_ and *P*_non-linear_ were from the generalized linear model, in which age, sex, duration of diabetes, BMI, TG, LDL-C, Matsuda index, insulin treatment, oral hypoglycemic agent treatment, ACE inhibitor/ARB treatment, and other antihypertensive agent treatment were adjusted. ISSI2, insulin secretion-sensitivity index-2; SBP, systolic blood pressure; HbA1c, glycosylated hemoglobin; BMI, body mass index; TG, triglyceride; LDL-C, low-density lipoprotein cholesterol; ACE, angiotensin converting enzyme; ARB, angiotensin receptor blocker

To further identify the optimal SBP level corresponding to the best beta-cell function, we performed RCS analyses in participants with HbA1c < 10% versus ≥ 10%, respectively. In participants with HbA1c ≥ 10%, we observed an inverted U-shape association between SBP and beta-cell function. Notably, ISSI2 progressively increased with increasing SBP reaching optimal at the SBP of 144 mmHg (difference between 144 mmHg and Ref. = 22.19, 95% CI 5.28–39.10), followed by reduction in ISSI2 at further increases in SBP (Fig. 1a). In contrast, in participants with HbA1c < 10%, there was neither a linear nor a non-linear association of the SBP with ISSI2 (Fig. 1b).

### Higher SBP was “paradoxically” associated with a lower 2 h PBG in participants with HbA1c ≥ 10%

**Fig. 2:**
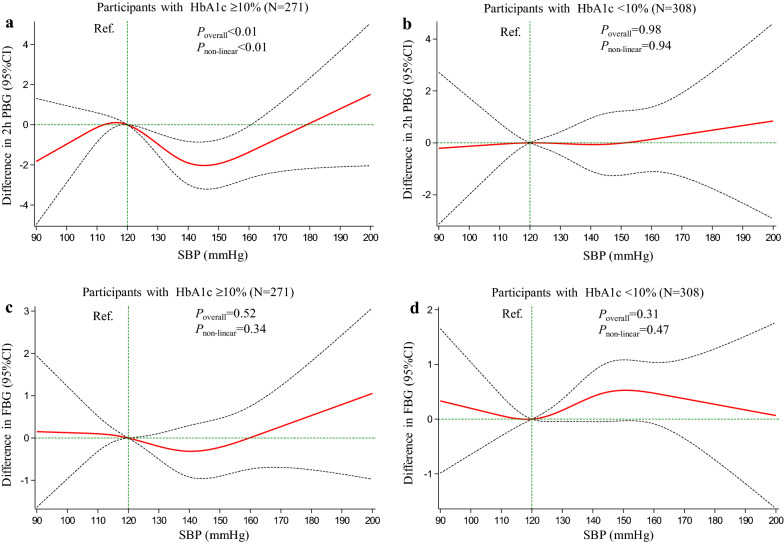
RCS analysis on the association of SBP with 2 h PBG (**a**, **b**) and FBG (**c**, **d**). *P*_overall_ and *P*_non-linear_ were from the generalized linear model, in which age, sex, duration of diabetes, BMI, TG, LDL-C, Matsuda index, insulin treatment, oral hypoglycemic agent treatment, ACE inhibitor/ARB treatment, and other antihypertensive agent treatment were adjusted. ISSI2, insulin secretion-sensitivity index-2; SBP, systolic blood pressure; PBG, postprandial blood glucose; FBG, fasting blood glucose; HbA1c, glycosylated hemoglobin; BMI, body mass index; TG, triglyceride; LDL-C, low-density lipoprotein cholesterol; ACE, angiotensin converting enzyme; ARB, angiotensin receptor blocker

The above results suggested that a certain degree of elevated SBP was associated with a better performing beta-cell function in participants with HbA1c ≥ 10%. We therefore next assessed whether an increasing SBP could improve blood glucose level, presumably resulting from the improved beta-cell function. We performed the RCS analysis on the association of SBP with 2 h PBG and also with FBG. In participants with HbA1c ≥ 10%, we found the SBP to be associated with the 2 h PBG, but in a non-linear pattern. Specifically, the 2 h PBG decreased as SBP increased from 120 to 145 mmHg (difference between 145 mmHg and Ref. = − 2.03 mmol/L, 95% CI − 3.20 to − 0.86), the 2 h PBG then tended to rise with further increases in SBP (Fig. 2a). In contrast, in participants with HbA1c < 10%, there was neither a linear nor a non-linear association of the SBP with the 2 h PBG (Fig. 2b). With FBG, there was also neither a linear nor non-linear association of the SBP with HbA1c ≥ 10% (Fig. 2c) or < 10% (Fig. 2d).

### Beta-cell function mediated the association between SBP and 2 h PBG in participants with HbA1c ≥ 10%

**Fig. 3:**
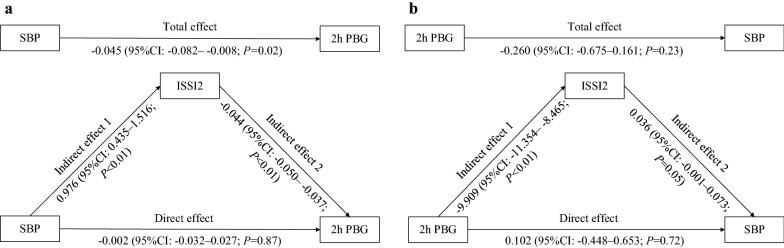
Mediating effect of islet beta-cell function. **a** effect of SBP on 2 h PBG. **b** effect of 2 h PBG on SBP. All analyses were performed in participants with HbA1c ≥ 10% and in the SBP < 150 mmHg. Mediating effect = Indirect effect 1 × Indirect effect 2. **a** N = 240 and adjusted age, sex, duration of diabetes, BMI, TG, LDL-C, Matsuda index, insulin treatment, oral hypoglycemic agent treatment, ACE inhibitor/ARB treatment, and other antihypertensive agent treatment. **b** N = 237 and adjusted age, sex, duration of diabetes, current smoking, current alcohol consumption, BMI, TG, TC, HDL-C, LDL-C, oral antihypertensive agent treatment, and self-reported hypertension. ISSI2, insulin secretion-sensitivity index-2; SBP, systolic blood pressure; PBG, postprandial blood glucose; HbA1c, glycosylated hemoglobin; BMI, body mass index; TG, triglyceride; TC, total cholesterol; HDL-C, high-density lipoprotein cholesterol; LDL-C, low-density lipoprotein cholesterol; ACE, angiotensin converting enzyme; ARB, angiotensin receptor blocker

The above results demonstrated that in participants with HbA1c ≥ 10%, SBP at less than 150 mmHg was associated with increased ISSI2 and decreased 2 h PBG. We then interrogated whether this association of the SBP and 2 h PBG was attributed to the postulated effects on beta-cell function by performing mediation analyses in participants with SBP < 150 mmHg. Mediation analyses showed that SBP was negatively associated with 2 h PBG before adjusting for ISSI2 (total effect = − 0.045, 95% CI − 0.082 to − 0.008) but not after adjusting for ISSI2 (direct effect = − 0.002, 95% CI − 0.032 to 0.027). The mediating effect of ISSI2 was − 0.043 (95% CI − 0.067 to − 0.018), which translates to indicate that beta-cell function might have mediated 94.7% (*P* < 0.01) of the association between SBP and 2 h PBG (Fig. 3a). To avoid reverse causality, we repeated the analysis with SBP as the dependent variable, 2 h PBG as the independent variable, and ISSI2 as the potential mediating variable. The direct effect of 2 h PBG on SBP was 0.102 (95% CI − 0.448 to 0.653) and the mediating effect of ISSI2 was − 0.359 (95% CI − 0.725 to 0.007), which indicated that 2 h PBG may not influence SBP and beta-cell function may not be a mediator in this analysis (Fig. 3b).

## Discussion

In this study, we demonstrated that a certain degree of elevated SBP, specifically 140–150 mmHg, was associated with better beta-cell function in participants with HbA1c ≥ 10% but not in those with HbA1c < 10%. This association was confirmed by the finding that beta-cell function mediated the “paradoxically” association of “higher blood pressure” and “lower 2 h PBG” in participants with HbA1c ≥ 10%. To the best of our knowledge, this is the first study to investigate the optimal range of SBP that would attain better islet beta-cell function in patients with T2DM.

We provided consistent evidence to support our conclusion that a slightly increased SBP (140–150 mmHg) rather than progressive increases in SBP contributed to the better beta-cell function in patients with HbA1c ≥ 10%. First, we demonstrated that the SBP of 140–150 mmHg group had the best beta-cell function among different SBP groups even after adjusting for important potential confounders (Table 2). Second, the robustness of this result was supported by performing sensitivity analyses in subjects with different characteristics (Additional file 1: Table S3). Third, the RCS analysis supported an inverted U-shape association between SBP and beta-cell function with the SBP at 144 mmHg corresponding to the best islet function (Fig. 1a). Notably, we further demonstrated that ISSI2 may mediate almost all of influence (94.7%) of SBP on 2 h PBG in participants with HbA1c ≥ 10% (Fig. 3). This finding in turn confirmed that a certain narrow range of high SBP may enhance beta-cell function and consequently reduce PBG.

Hyperglycemia has a direct effect on increasing blood viscosity [4, 6]. Additionally, hyperglycemia could trigger osmotic diuresis to increase blood hematocrit, plasma fibrinogen level and reduce plasma volume, all of which contribute to higher blood viscosity [5, 24]. To overcome perfusion resistance resulting from the increased blood viscosity, an increase in BP might be a compensatory mechanism to ensure adequate blood supply to vital organs [4]. Increase in blood glucose concentration from 100 to 400 mg/dL in patients with T2DM led to a 25% increase in blood viscosity and a 20% decrease in blood flow rate. Accordingly, BP had to increase by 25% to compensate for the reduced blood flow rate [4]. Moreover, the studies conducted in diabetic and non-diabetic individuals also confirmed that BP elevated along with increases in blood viscosity [8, 9, 25]. These studies taken together could explain the reason for the association of higher BP and better beta-cell function. Indeed, the compensatory effect of a higher BP has been observed in patients with acute primary intracerebral hemorrhage. Compared to those with the BP target of SBP < 140 mmHg, a higher BP target (SBP < 160 mmHg) was associated with decreased remote cerebral ischemic lesions, acute neurologic deterioration, and shorter health-care time in hospital since the higher BP presumably reduces brain tissue ischemia [26].

As for why BP was only associated with PBG but not with FBG in participants with HbA1c ≥ 10%, this might be related to the complex regulation of blood glucose. Glucose-stimulated insulin secretion (GSIS) mainly occurs in the post-prandial state [27], wherein the pancreatic islets acutely secrete large amounts of insulin at full peak capacity [28]. However, due to the loss of the early phase of insulin secretion in patients with T2DM [28], PBG would rise rapidly to cause a remarkable increase in blood viscosity, which reduces the perfusion of the pancreas and consequently inhibits beta-cell function [6]. In this situation, a higher SBP level can overcome the perfusion resistance and maintain blood supply that would maintain beta-cells in good function [1]. In contrast, FBG is usually significantly lower than PBG, and FBG is mainly regulated by basal insulin secretion, whose level and secretion rate are significantly lower than that of GSIS [28, 29]. Thus, in the fasting state, islet cells might be able to regulate FBG without mobilizing all insulin secretory capacity nor the compensatory enhancement from high BP.

We demonstrated that elevated BP was associated with better beta-cell function in T2DM patients with the duration of diabetes < 1 year rather than in those with the duration of diabetes ≥ 1 year (Additional file 1: Table S3), which was consistent with a previous study [10]. This study investigated the factors that can influence beta-cell function in patients with newly diagnosed T2DM and found that a higher level of SBP was associated with an improved beta-cell function [10]. However, our study showed this positive association only occurred in an increased but narrow SBP range of 140–150 mmHg, and not with further increases in SBP beyond this range.

This study therefore raises the possibility that transiently elevated BP may not always have deleterious effect on patients with early T2DM, especially when they have poor glycemic control. Short-term elevation in SBP might be a compensatory mechanism in response to hyperglycemia-induced increase in blood viscosity, which aims to ensure sufficient blood supply to vital organs, in this case, the pancreatic islets. We should nevertheless recognize that hypertension is a major risk factor for cardiovascular disease [30], which is the leading cause of morbidity and mortality for individuals with diabetes [31–33]. Thus, we must highlight that our study does not encourage the maintenance of a sustained state of hypertension in T2DM patients with poor glycemic control. Maintaining a blood pressure and blood glucose level within appropriate ranges remains to be the recommended strategy for diabetes management.

This study has two strengths. First, we diminished the influence of oral antihypertensive agents, insulin treatment, and duration of diabetes on islet beta-cell function by performing analyses only on subjects without taking oral antihypertensive agents, without accepting insulin treatment, and with duration of diabetes < 1 year, respectively. Second, we confirmed that higher SBP may have a favorable effect on beta-cell function by demonstrating that beta-cell function mediated the influence of SBP on 2 h PBG but not a reverse causality. Nevertheless, there remain some limitations. First, the cross-sectional study design is not warranted to infer causal relationships between SBP and beta-cell function. Second, the sample size in some SBP groups is relatively small. More prospective studies with large sample size are needed to verify the relationship between elevated BP and islet beta-cell function. Third, the possibility of spurious association findings still cannot be ruled out due to the exploratory nature of the study, multiple dichotomies of input parameters, and multiple testing. Fourth, the heterogeneity of the patient population studied, especially regarding hemodynamic status and treatments, may bias data interpretation through residual confounders despite adjustments. Finally, the dissociation of the findings that SBP rather than DBP was associated with beta-cell function may weaken the proposed pathophysiological hypothesis. However, clinical data have shown that SBP, not DBP, was positively associated with beta-cell function [10, 11]. Organ perfusion is more vulnerable to changes in SBP than DBP [34]. Thus, more studies are desired to elucidate the pathophysiological mechanisms underlying the effects of BP on beta-cell function.

## Conclusions

A slight elevation in SBP (140–150 mmHg) was associated with better beta-cell function and reduced 2 h PBG in T2DM patients with HbA1c ≥ 10% with a duration of diabetes of < 1 year. This study adds new knowledge to our understanding that during the progression of T2DM, there might be a unique period in which a slightly elevated SBP may enhance islet beta-cell function.

## Supplementary Information

**Additional file 1**: Additional Tables.

## Data Availability

The datasets used and/or analyzed during the current study are available from the corresponding author on reasonable request.

## Abbreviations

BP: Blood pressure
T2DM: Type 2 diabetes mellitus
HbA1c: Glycosylated hemoglobin
SBP: Systolic blood pressure
DBP: Diastolic blood pressure
SBMT: Steamed bread meal test
ISSI2: Insulin secretion-sensitivity index-2
HOMA2-IR: Homeostatic model assessment 2-insulin resistance
HOMA2-B: Homeostatic model assessment 2-beta
TG: Triglyceride
LDL-C: Low-density lipoprotein cholesterol
TC: Total cholesterol
HDL-C: High-density lipoprotein cholesterol
RCS: Restricted cubic splines
PBG: Postprandial blood glucose
FBG: Fasting blood glucose
GSIS: Glucose-stimulated insulin secretion
ACE: Angiotensin converting enzyme
ARB: Angiotensin receptor blocker
