# Supplementary material for: Higher systolic blood pressure is specifically associated with better islet beta-cell function in T2DM patients with high glycemic level
Source: Cardiovasc Diabetol. 2022 Dec 19;21:283. doi: 10.1186/s12933-022-01723-1 (PMC9764532; doi:10.1186/s12933-022-01723-1)
Supplement: Supplementary file 1 — Additional file 1: Additional Tables. [file 12933_2022_1723_MOESM1_ESM.docx]

**Additional file 1**

| Table S1 Demographic and clinical characteristics of participants among different SBP groups | | | | | | |
| --- | --- | --- | --- | --- | --- | --- |
| Characteristics | 90≤SBP<120, mmHg | 120≤SBP<130, mmHg | 130≤SBP<140, mmHg | 140≤SBP<150, mmHg | 150≤SBP, mmHg | *P* value |
| Participants with HbA1c <10% | |  |  |  |  |  |
| N | 76 | 65 | 73 | 53 | 53 |  |
| Age, mean ± SD, year | 48.5 ± 9.3 | 53.4 ± 11.3 | 49.9 ± 11.5 | 52.8 ± 11.7 | 54.4 ± 13.1 | 0.01 |
| Male sex, n (%) | 44 (57.9) | 42 (64.6) | 52 (71.2) | 34 (64.2) | 34 (64.2) | 0.58 |
| Current smoking, n (%) | 19 (25.0) | 18 (28.1) | 24 (33.3) | 17 (32.1) | 17 (32.1) | 0.81 |
| Current alcohol consumption, n (%) | 30 (39.5) | 27 (42.2) | 31 (43.1) | 27 (50.9) | 23 (43.4) | 0.78 |
| Duration of diabetes, median (25th–75th), year | 4.4 (0.7–9.2) | 7.0 (1.5–10.6) | 5.0 (1.1–10.0) | 6.2 (2.0–13.6) | 6.3 (2.0–12.3) | 0.30 |
| Hypertension, n (%) | 15 (19.7) | 25 (38.5) | 45 (61.6) | 53 (100.0) | 53 (100.0) | <0.01 |
| Self-reported hypertension, n (%) | 11 (14.5) | 15 (23.1) | 30 (41.1) | 26 (49.1) | 32 (60.4) | <0.01 |
| Newly diagnosed hypertension, n (%) | 4 (5.3) | 10 (15.4) | 15 (20.6) | 27 (50.9) | 21 (39.6) | <0.01 |
| CHD, n (%) | 14 (18.4) | 16 (24.6) | 12 (16.4) | 10 (18.9) | 13 (24.5) | 0.69 |
| Stroke, n (%) | 7 (9.2) | 6 (9.2) | 8 (11.0) | 7 (13.2) | 13 (24.5) | 0.08 |
| BMI, mean ± SD, kg/m^2^ | 24.7 ± 2.2 | 25.3 ± 3.4 | 27.0 ± 4.3 | 27.2 ± 3.9 | 26.4 ± 5.0 | <0.01 |
| SBP, mean ± SD, mmHg | 111.8 ± 6.1 | 124.6 ± 2.8 | 134.2 ± 3 | 144.5 ± 3 | 160.5 ± 9.2 | <0.01 |
| DBP, mean ± SD, mmHg | 71.8 ± 6.2 | 77.2 ± 7.2 | 82.9 ± 7.8 | 87.2 ± 8.8 | 91.9 ± 9.8 | <0.01 |
| HbA1c, mean ± SD, % | 8.0 ± 1.3 | 8.1 ± 1.1 | 8.2 ± 1.2 | 8.3 ± 1.1 | 8.3 ± 1.2 | 0.68 |
| HbA1c, mean ± SD, mmol/mol | 64.1 ± 14.0 | 65.2 ± 12.0 | 66.5 ± 12.8 | 66.8 ± 11.5 | 66.7 ± 13.0 | 0.68 |
| FBG, mean ± SD, mmol/L | 7.3 ± 1.9 | 7.7 ± 1.9 | 7.4 ± 1.8 | 8.1 ± 2.4 | 7.9 ± 1.8 | 0.15 |
| 2h PBG, mean ± SD, mmol/L | 16.1 ± 4.6 | 16.2 ± 4.4 | 15.6 ± 4.3 | 15.6 ± 4.9 | 16.7 ± 4.6 | 0.71 |
| TG, median (25th–75th), mmol/L | 1.3 (0.9–2.2) | 1.5 (1.1–3.0) | 1.7 (1.1–2.0) | 1.7 (1.1–2.6) | 1.7 (1.1–2.7) | 0.17 |
| TC, median (25th–75th), mmol/L | 4.1 (3.7–4.8) | 4.4 (3.8–5.1) | 4.5 (3.9–5.2) | 4.6 (3.8–5.1) | 4.4 (3.5–5.2) | 0.34 |
| HDL-C, median (25th–75th), mmol/L | 0.9 (0.8–1.3) | 1.0 (0.8–1.2) | 1.0 (0.8–1.2) | 1.0 (0.9–1.2) | 1.0 (0.8–1.2) | 1.00 |
| LDL-C, median (25th–75th), mmol/L | 2.4 (1.9–2.9) | 2.6 (2.1–3.0) | 2.6 (2.2–3.2) | 2.5 (1.9–3.1) | 2.3 (1.7–3.0) | 0.22 |
| ALT, median (25th–75th), U/L | 17.8 (13.3–28.5) | 17.4 (12.9–24.9) | 24.7 (15.2–47.7) | 18.5 (12.9–30.2) | 14.7 (11.7–29.1) | 0.01 |
| AST, median (25th–75th), U/L | 16.0 (13.4–21.5) | 14.7 (12.3–19.5) | 18.6 (14.2–26.9) | 15.9 (14.0–22.0) | 17.0 (13.0–22.0) | 0.05 |
| UA, median (25th–75th), μmol/L | 286.0 (246.5–329.0) | 269.0 (234.0–356.0) | 310.5 (255.0–366.0) | 284.0 (245.0–332.0) | 338.0 (251.0–381.0) | 0.06 |
| Oral antihypertensive agent treatment  (not mutually exclusive below), n (%) | 13 (17.1) | 21 (32.3) | 24 (32.9) | 26 (49.1) | 26 (49.1) | <0.01 |
| Calcium channel blocker, n (%) | 8 (10.5) | 7 (10.8) | 13 (17.8) | 13 (24.5) | 16 (30.2) | 0.02 |
| ACE inhibitor/ARB, n (%) | 10 (13.2) | 9 (13.9) | 20 (27.4) | 16 (30.2) | 24 (45.3) | <0.01 |
| Alpha blocker/Beta blocker, n (%) | 8 (10.5) | 14 (21.5) | 12 (16.4) | 12 (22.6) | 11 (20.8) | 0.33 |
| Diuretic, n (%) | 1 (1.3) | 3 (4.6) | 6 (8.2) | 7 (13.2) | 11 (20.8) | <0.01 |
| Insulin treatment, n (%) | 17 (22.4) | 14 (21.5) | 17 (23.3) | 14 (26.4) | 18 (34.0) | 0.54 |
| Oral hypoglycemic agent treatment, n (%) | 53 (69.7) | 54 (83.1) | 52 (71.2) | 38 (71.7) | 39 (73.6) | 0.42 |
| HOMA2-IR, median (25th–75th) | 1.7 (1.2–2.2) | 1.8 (1.3–2.1) | 2.0 (1.5–2.8) | 1.8 (1.4–2.3) | 2.0 (1.4–2.5) | 0.11 |
| Matsuda index, median (25th–75th) | 77.8 (50.5–129.8) | 82.1 (61.5–122.7) | 65.9 (37.6–98.1) | 81.7 (51.4–106.8) | 63.8 (38.9–92.1) | 0.03 |
| HOMA2-B, median (25th–75th) | 67.4 (42.5–100.6) | 57.5 (42.5–81.2) | 68.6 (46.7–117.7) | 64.5 (38.8–91.3) | 60.2 (40.5–79.0) | 0.07 |
| ISSI2, median (25th–75th) | 147.9 (92.4–211.0) | 143.0 (84.9–193.4) | 145.5 (108.0–211.6) | 137.5 (86.3–185.8) | 109.2 (85.5–150.5) | 0.31 |
| Participants with HbA1c ≥10% | |  |  |  |  |  |
| N | 91 | 66 | 59 | 36 | 31 |  |
| Age, mean ± SD, year | 44.6 ± 11.6 | 45.6 ± 11.1 | 47.4 ± 13.0 | 47.8 ± 13.9 | 49.3 ± 12.9 | 0.32 |
| Male sex, n (%) | 67 (73.6) | 49 (74.2) | 42 (71.2) | 23 (63.9) | 16 (51.6) | 0.15 |
| Current smoking, n (%) | 40 (44.0) | 23 (34.9) | 21 (35.6) | 9 (25.7) | 9 (29.0) | 0.31 |
| Current alcohol consumption, n (%) | 49 (53.9) | 37 (56.1) | 25 (42.4) | 18 (51.4) | 10 (32.3) | 0.15 |
| Duration of diabetes, median (25th–75th), year | 0.1 (0.0–3.5) | 1.4 (0.1–7.0) | 1.0 (0.1–7.0) | 1.2 (0.0–8.2) | 7.0 (0.7–12.3) | <0.01 |
| Hypertension, n (%) | 13 (14.3) | 18 (27.3) | 25 (42.4) | 36 (100.0) | 31 (100.0) | <0.01 |
| Self-reported hypertension, n (%) | 10 (11.0) | 15 (22.7) | 17 (28.8) | 16 (44.4) | 20 (64.5) | <0.01 |
| Newly diagnosed hypertension, n (%) | 3 (3.3) | 3 (4.6) | 8 (13.6) | 20 (55.6) | 11 (35.5) | <0.01 |
| CHD, n (%) | 3 (3.3) | 3 (4.6) | 6 (10.2) | 5 (13.9) | 7 (22.6) | <0.01 |
| Stroke, n (%) | 2 (2.2) | 3 (4.6) | 5 (8.5) | 2 (5.6) | 6 (19.4) | 0.02 |
| BMI, mean ± SD, kg/m^2^ | 24.7 ± 3.8 | 26.0 ± 3.1 | 25.8 ± 3.2 | 26.9 ± 3.7 | 27.0 ± 3.6 | <0.01 |
| SBP, mean ± SD, mmHg | 111.9 ± 5.9 | 124.3 ± 2.9 | 134.5 ± 2.9 | 144.0 ± 3.0 | 163.1 ± 10.3 | <0.01 |
| DBP, mean ± SD, mmHg | 72.7 ± 6.4 | 79.7 ± 6.8 | 82.7 ± 8.4 | 85.4 ± 9.3 | 94.5 ± 12.6 | <0.01 |
| HbA1c, mean ± SD, % | 11.9 ± 1.2 | 11.6 ± 1.5 | 11.5 ± 1.3 | 11.3 ± 1.2 | 11.5 ± 1.3 | 0.10 |
| HbA1c, mean ± SD, mmol/mol | 107.0 ± 13.6 | 102.9 ± 16.7 | 102.7 ± 14.1 | 100.0 ± 13.4 | 102.3 ± 13.7 | 0.10 |
| FBG, mean ± SD, mmol/L | 8.7 ± 2.0 | 8.8 ± 2.7 | 9.1 ± 2.2 | 9.0 ± 3.2 | 9.4 ± 2.7 | 0.71 |
| 2h PBG, mean ± SD, mmol/L | 18.7 ± 3.9 | 18.0 ± 4.4 | 17.6 ± 4.8 | 17.1 ± 5.3 | 17.9 ± 4.4 | 0.40 |
| TG, median (25th–75th), mmol/L | 1.3 (0.9–2.2) | 1.9 (1.1–3.7) | 1.5 (1.0–3.7) | 1.5 (1.1–2.5) | 1.8 (1.2–2.9) | 0.24 |
| TC, median (25th–75th), mmol/L | 4.6 (3.9–5.2) | 4.7 (4.1–5.6) | 4.8 (4.0–5.4) | 4.7 (4.1–5.7) | 4.9 (4.0–5.6) | 0.60 |
| HDL-C, median (25th–75th), mmol/L | 1.0 (0.8–1.2) | 1.0 (0.8–1.2) | 1.0 (0.8–1.2) | 1.0 (0.8–1.1) | 1.0 (0.9–1.1) | 0.89 |
| LDL-C, median (25th–75th), mmol/L | 2.8 (2.4–3.4) | 2.6 (2.2–3.3) | 2.6 (2.1–3.3) | 2.9 (2.3–3.7) | 2.8 (2.0–3.5) | 0.72 |
| ALT, median (25th–75th), U/L | 18.2 (11.5–27.0) | 20.1 (14.7–33.4) | 19.7 (14.0–25.5) | 22.1 (13.9–30.5) | 17.7 (12.7–29.2) | 0.64 |
| AST, median (25th–75th), U/L | 14.1 (11.4–19.0) | 16.4 (12.0–21.1) | 15.7 (14.0–20.6) | 15.3 (12.0–23.9) | 14.6 (11.3–21.5) | 0.20 |
| UA, median (25th–75th), μmol/L | 262.0 (212.0–332.0) | 291.0 (248.0–362.0) | 262.5 (216.5–323.0) | 277.0 (213.0–334.0) | 263.5 (208.5–326.0) | 0.26 |
| Oral antihypertensive agent treatment  (not mutually exclusive below), n (%) | 8 (8.8) | 10 (15.2) | 9 (15.3) | 11 (30.6) | 17 (54.8) | <0.01 |
| Calcium channel blocker, n (%) | 4 (4.4) | 6 (9.1) | 5 (8.5) | 7 (19.4) | 11 (35.5) | <0.01 |
| ACE inhibitor/ARB, n (%) | 6 (6.6) | 7 (10.6) | 11 (18.6) | 9 (25.0) | 13 (41.9) | <0.01 |
| Alpha blocker/Beta blocker, n (%) | 5 (5.5) | 2 (3.0) | 4 (6.8) | 3 (8.3) | 7 (22.6) | 0.03 |
| Diuretic, n (%) | 1 (1.1) | 5 (7.6) | 2 (3.4) | 5 (13.9) | 7 (22.6) | <0.01 |
| Insulin treatment, n (%) | 9 (9.9) | 15 (22.7) | 13 (22.0) | 6 (16.7) | 11 (35.5) | 0.02 |
| Oral hypoglycemic agent treatment, n (%) | 32 (35.2) | 35 (53.0) | 32 (54.2) | 18 (50.0) | 20 (64.5) | 0.03 |
| HOMA2-IR, median (25th–75th) | 1.5 (1.2–1.8) | 1.7 (1.4–2.2) | 1.8 (1.5–2.2) | 1.8 (1.5–2.4) | 1.9 (1.5–2.6) | <0.01 |
| Matsuda index, median (25th–75th) | 106.6 (76.6–156.5) | 87.9 (59.5–132.3) | 91.5 (58.7–120.4) | 75.4 (52.3–104.8) | 67.0 (50.1–105.2) | <0.01 |
| HOMA2-B, median (25th–75th) | 40.6 (29.3–52.3) | 46.0 (32.0–72.3) | 45.3 (30.4–56.8) | 50.6 (33.6–83.5) | 50.9 (31.7–68.3) | 0.11 |
| ISSI2, median (25th–75th) | 90.7 (67.6–127.9) | 106.1 (72.0–149.5) | 96.1 (64.6–130.4) | 106.5 (64.4–180.8) | 91.8 (60.2–132.8) | 0.62 |
| *P* value was from one-way ANOVA, Kruskal-Wallis H test, or Chi-squared test. CHD, coronary heart disease; BMI, body mass index; SBP, systolic blood pressure; DBP, diastolic blood pressure; HbA1c, glycosylated hemoglobin; FBG, fasting blood glucose; PBG, postprandial blood glucose; TG, triglyceride; TC, total cholesterol; HDL-C, high-density lipoprotein cholesterol; LDL-C, low-density lipoprotein cholesterol; ALT, alanine transaminase; AST, aspartate aminotransferase; UA, uric acid; ACE, angiotensin converting enzyme; ARB, angiotensin receptor blocker; HOMA2-IR, homeostatic model assessment 2-insulin resistance; HOMA2-B, homeostatic model assessment 2-beta; ISSI2, insulin secretion-sensitivity index-2. | | | | | | |

| Table S2 Demographic and clinical characteristics of participants among different DBP groups | | | | |
| --- | --- | --- | --- | --- |
| Characteristics | 60≤DBP<80, mmHg | 80≤DBP<90, mmHg | 90≤DBP, mmHg | *P* value |
| Participants with HbA1c <10% | |  |  |  |
| N | 150 | 94 | 76 |  |
| Age, mean ± SD, year | 52.9 ± 11.1 | 51.1 ± 12.5 | 49.1 ± 10.3 | 0.05 |
| Male sex, n (%) | 85 (56.7) | 66 (70.2) | 55 (72.4) | 0.02 |
| Current smoking, n (%) | 38 (25.3) | 30 (32.6) | 27 (35.5) | 0.23 |
| Current alcohol consumption, n (%) | 55 (36.7) | 41 (44.6) | 42 (55.3) | 0.03 |
| Duration of diabetes, median (25th–75th), year | 6.9 (2.1–11.0) | 5.5 (1.0–10.6) | 3.2 (0.5–9.5) | 0.04 |
| Hypertension, n (%) | 59 (39.3) | 56 (59.6) | 76 (100.0) | <0.01 |
| Self-reported hypertension, n (%) | 38 (25.3) | 39 (41.5) | 37 (48.7) | <0.01 |
| Newly diagnosed hypertension, n (%) | 21 (14.0) | 17 (18.1) | 39 (51.3) | <0.01 |
| CHD, n (%) | 37 (24.7) | 18 (19.2) | 10 (13.2) | 0.12 |
| Stroke, n (%) | 18 (12.0) | 14 (14.9) | 9 (11.8) | 0.77 |
| BMI, mean ± SD, kg/m^2^ | 25.4 ± 3.0 | 26.4 ± 4.8 | 26.9 ± 4.0 | 0.02 |
| SBP, mean ± SD, mmHg | 123.1 ± 13.9 | 136.1 ± 13.7 | 148.7 ± 13.8 | <0.01 |
| DBP, mean ± SD, mmHg | 72.4 ± 5.2 | 84.0 ± 2.9 | 95.7 ± 5.5 | <0.01 |
| HbA1c, mean ± SD, % | 8.1 ± 1.2 | 8.2 ± 1.1 | 8.2 ± 1.2 | 0.91 |
| HbA1c, mean ± SD, mmol/mol | 65.5 ± 13.0 | 65.7 ± 12.2 | 66.3 ± 12.9 | 0.91 |
| FBG, mean ± SD, mmol/L | 7.8 ± 2.1 | 7.5 ± 1.9 | 7.6 ± 1.8 | 0.48 |
| 2h PBG, mean ± SD, mmol/L | 16.5 ± 4.8 | 16.0 ± 4.4 | 15.2 ± 4.3 | 0.13 |
| TG, median (25th–75th), mmol/L | 1.4 (0.9–2.2) | 1.5 (1.0–2.3) | 1.8 (1.2–3.2) | 0.12 |
| TC, median (25th–75th), mmol/L | 4.3 (3.7–5.2) | 4.4 (3.8–4.9) | 4.7 (3.8–5.2) | 0.44 |
| HDL-C, median (25th–75th), mmol/L | 1.0 (0.8–1.2) | 0.9 (0.8–1.2) | 1.0 (0.8–1.2) | 0.88 |
| LDL-C, median (25th–75th), mmol/L | 2.5 (1.9–3.0) | 2.6 (2.0–3.1) | 2.4 (1.9–3.2) | 0.85 |
| ALT, median (25th–75th), U/L | 17.6 (12.7–28.0) | 20.0 (14.2–32.6) | 19.7 (13.5–29.4) | 0.12 |
| AST, median (25th–75th), U/L | 16.1 (13.4–21.3) | 16.7 (13.0–23.5) | 16.6 (14.4–22.3) | 0.56 |
| UA, median (25th–75th), μmol/L | 272.0 (234.0–343.0) | 320.5 (255.0–371.0) | 309.0 (251.0–355.0) | <0.01 |
| Oral antihypertensive agent treatment  (not mutually exclusive below), n (%) | 43 (28.7) | 33 (35.1) | 34 (44.7) | 0.05 |
| Calcium channel blocker, n (%) | 20 (13.3) | 15 (16.0) | 22 (29.0) | 0.01 |
| ACE inhibitor/ARB, n (%) | 26 (17.3) | 26 (27.7) | 27 (35.5) | <0.01 |
| Alpha blocker/Beta blocker, n (%) | 26 (17.3) | 17 (18.1) | 14 (18.4) | 0.98 |
| Diuretic, n (%) | 7 (4.7) | 11 (11.7) | 10 (13.2) | 0.05 |
| Insulin treatment, n (%) | 39 (26.0) | 25 (26.6) | 16 (21.1) | 0.66 |
| Oral hypoglycemic agent treatment, n (%) | 115 (76.7) | 69 (73.4) | 52 (68.4) | 0.41 |
| HOMA2-IR, median (25th–75th) | 1.8 (1.3–2.2) | 1.8 (1.3–2.4) | 1.9 (1.4–2.5) | 0.44 |
| Matsuda index, median (25th–75th) | 75.7 (50.5–106.0) | 71.9 (45.2–112.5) | 69.7 (43.6–97.6) | 0.56 |
| HOMA2-B, median (25th–75th) | 61.3 (39.1–90.7) | 66.8 (47.2–95.9) | 66.6 (42.8–97.9) | 0.31 |
| ISSI2, median (25th–75th) | 140.0 (83.6–197.7) | 134.0 (87.0–187.5) | 147.3 (99.3–208.1) | 0.56 |
| Participants with HbA1c ≥10% | |  |  |  |
| N | 143 | 91 | 49 |  |
| Age, mean ± SD, year | 46.7 ± 12.6 | 46.5 ± 12.0 | 45.1 ± 11.9 | 0.75 |
| Male sex, n (%) | 94 (65.7) | 67 (73.6) | 36 (73.5) | 0.36 |
| Current smoking, n (%) | 51 (35.7) | 36 (39.6) | 15 (31.3) | 0.62 |
| Current alcohol consumption, n (%) | 68 (47.6) | 48 (52.8) | 23 (47.9) | 0.72 |
| Duration of diabetes, median (25th–75th), year | 1.0 (0.1–7.2) | 0.3 (0.1–7.0) | 2.9 (0.1–7.0) | 0.37 |
| Hypertension, n (%) | 40 (28.0) | 34 (37.4) | 49 (100.0) | <0.01 |
| Self-reported hypertension, n (%) | 30 (21.0) | 24 (26.4) | 24 (49.0) | <0.01 |
| Newly diagnosed hypertension, n (%) | 10 (7.0) | 10 (11.0) | 25 (51.0) | <0.01 |
| CHD, n (%) | 11 (7.7) | 8 (8.8) | 5 (10.2) | 0.86 |
| Stroke, n (%) | 10 (7.0) | 3 (3.3) | 5 (10.2) | 0.25 |
| BMI, mean ± SD, kg/m^2^ | 25.1 ± 3.6 | 26.0 ± 3.4 | 27.2 ± 3.5 | <0.01 |
| SBP, mean ± SD, mmHg | 121.6 ± 14.0 | 132.1 ± 14.2 | 145.9 ± 16.5 | <0.01 |
| DBP, mean ± SD, mmHg | 72.3 ± 5.0 | 83.8 ± 2.8 | 97.7 ± 7.1 | <0.01 |
| HbA1c, mean ± SD, % | 11.7 ± 1.3 | 11.5 ± 1.4 | 11.6 ± 1.4 | 0.40 |
| HbA1c, mean ± SD, mmol/mol | 104.8 ± 14.0 | 102.1 ± 15.2 | 103.8 ± 15.0 | 0.40 |
| FBG, mean ± SD, mmol/L | 9.1 ± 2.4 | 9.0 ± 2.6 | 8.5 ± 2.3 | 0.36 |
| 2h PBG, mean ± SD, mmol/L | 18.6 ± 4.5 | 17.5 ± 4.4 | 17.2 ± 4.2 | 0.06 |
| TG, median (25th–75th), mmol/L | 1.3 (0.9–2.4) | 1.5 (1.1–3.7) | 2.1 (1.2–3.0) | <0.01 |
| TC, median (25th–75th), mmol/L | 4.5 (3.8–5.2) | 4.8 (4.2–5.7) | 5.1 (4.5–5.7) | <0.01 |
| HDL-C, median (25th–75th), mmol/L | 1.0 (0.8–1.2) | 1.0 (0.8–1.1) | 1.0 (0.8–1.2) | 0.84 |
| LDL-C, median (25th–75th), mmol/L | 2.8 (2.2–3.4) | 2.7 (2.1–3.6) | 2.9 (2.2–3.5) | 0.72 |
| ALT, median (25th–75th), U/L | 18.2 (12.7–27.7) | 18.1 (13.9–27.9) | 22.5 (14.4–35.7) | 0.30 |
| AST, median (25th–75th), U/L | 15.0 (11.8–20.0) | 15.1 (12.3–19.8) | 16.0 (12.5–22.3) | 0.46 |
| UA, median (25th–75th), μmol/L | 268.0 (212.0–327.0) | 284.0 (220.0–345.0) | 286.0 (213.0–333.5) | 0.55 |
| Oral antihypertensive agent treatment  (not mutually exclusive below), n (%) | 24 (16.8) | 15 (16.5) | 16 (32.7) | 0.04 |
| Calcium channel blocker, n (%) | 13 (9.1) | 9 (9.9) | 11 (22.5) | 0.03 |
| ACE inhibitor/ARB, n (%) | 16 (11.2) | 17 (18.7) | 13 (26.5) | 0.03 |
| Alpha blocker/Beta blocker, n (%) | 8 (5.6) | 5 (5.5) | 8 (16.3) | 0.03 |
| Diuretic, n (%) | 10 (7.0) | 5 (5.5) | 5 (10.2) | 0.58 |
| Insulin treatment, n (%) | 26 (18.2) | 15 (16.5) | 13 (26.5) | 0.33 |
| Oral hypoglycemic agent treatment, n (%) | 67 (46.9) | 45 (49.5) | 25 (51.0) | 0.86 |
| HOMA2-IR, median (25th–75th) | 1.6 (1.3–2.0) | 1.7 (1.3–2.4) | 1.8 (1.5–2.5) | 0.02 |
| Matsuda index, median (25th–75th) | 94.6 (69.7–134.7) | 79.8 (54.7–121.1) | 79.0 (63.7–137.6) | 0.11 |
| HOMA2-B, median (25th–75th) | 40.2 (29.3–51.3) | 47.5 (30.4–65.4) | 53.6 (37.1–77.8) | <0.01 |
| ISSI2, median (25th–75th) | 89.7 (65.2–127.9) | 97.6 (63.6–140.6) | 114.8 (81.2–146.2) | 0.08 |
| *P* value was from one-way analysis of variance, Kruskal-Wallis H test, or Chi-square test. CHD, coronary heart disease; BMI, body mass index; SBP, systolic blood pressure; DBP, diastolic blood pressure; HbA1c, glycosylated hemoglobin; FBG, fasting blood glucose; PBG, postprandial blood glucose; TG, triglyceride; TC, total cholesterol; HDL-C, high-density lipoprotein cholesterol; LDL-C, low-density lipoprotein cholesterol; ALT, alanine transaminase; AST, aspartate aminotransferase; UA, uric acid; ACE, angiotensin converting enzyme; ARB, angiotensin receptor blocker; HOMA2-IR, homeostatic model assessment 2-insulin resistance; HOMA2-B, homeostatic model assessment 2-beta; ISSI2, insulin secretion-sensitivity index-2. | | | | |

| Table S3 Sensitivity analysis on the association between BP and ISSI2 in participants with HbA1c ≥10% | | | |
| --- | --- | --- | --- |
| Factor | N | ISSI2 | *b*(95%CI) |
| SBP model |  |  |  |
| Subjects with insulin sensitivity in 10th-90th percentile^*^ | | |  |
| 90≤SBP<120, mmHg | 68 | 87.3 (67.2–127.1) | Ref. |
| 120≤SBP<130, mmHg | 49 | 102.0 (78.1–146.2) | 0.148 (-0.015–0.311) |
| 130≤SBP<140, mmHg | 45 | 91.5 (64.6–129.9) | 0.108 (-0.060–0.275) |
| 140≤SBP<150, mmHg | 29 | 104.3 (65.3–169.8) | 0.205 (0.012–0.399) |
| 150≤SBP, mmHg | 24 | 96.5 (65.8–129.4) | 0.088 (-0.140–0.317) |
| Subjects without taking oral antihypertensive agents^†^ | | |  |
| 90≤SBP<120, mmHg | 80 | 90.2 (67.5–128.9) | Ref. |
| 120≤SBP<130, mmHg | 55 | 111.6 (71.5–149.5) | 0.123 (-0.027–0.273) |
| 130≤SBP<140, mmHg | 45 | 98.0 (68.2–145.2) | 0.179 (0.016–0.342) |
| 140≤SBP<150, mmHg | 25 | 112.6 (77.8–190.6) | 0.321 (0.122–0.520) |
| 150≤SBP, mmHg | 14 | 118.2 (66.5–131.2) | 0.142 (-0.118–0.402) |
| Subjects without accepting insulin treatment^‡^ | | |  |
| 90≤SBP<120, mmHg | 81 | 91.7 (74.2–130.0) | Ref. |
| 120≤SBP<130, mmHg | 51 | 120.2 (81.7–164.1) | 0.148 (-0.002–0.298) |
| 130≤SBP<140, mmHg | 42 | 98.8 (68.2–135.2) | 0.110 (-0.053–0.274) |
| 140≤SBP<150, mmHg | 30 | 112.8 (77.8–190.6) | 0.281 (0.095–0.466) |
| 150≤SBP, mmHg | 20 | 119.7 (80.1–142.9) | 0.114 (-0.109–0.337) |
| Subjects with the duration of diabetes <1 year^§^ | | |  |
| 90≤SBP<120, mmHg | 55 | 99.3 (76.4–133.9) | Ref. |
| 120≤SBP<130, mmHg | 30 | 114.9 (86.2–167.7) | 0.171 (-0.033–0.375) |
| 130≤SBP<140, mmHg | 21 | 77.5 (62.9–145.2) | 0.091 (-0.153–0.335) |
| 140≤SBP<150, mmHg | 16 | 159.1 (95.2–293.9) | 0.354 (0.086–0.623) |
| 150≤SBP, mmHg | 8 | 119.1 (96.5–138.3) | 0.190 (-0.194–0.574) |
| Subjects with the duration of diabetes ≥1 year^§^ | | |  |
| 90≤SBP<120, mmHg | 32 | 79.0 (60.2–121.3) | Ref. |
| 120≤SBP<130, mmHg | 34 | 107.0 (55.0–136.5) | 0.093 (-0.115–0.302) |
| 130≤SBP<140, mmHg | 33 | 98.0 (64.6–130.4) | 0.115 (-0.092–0.323) |
| 140≤SBP<150, mmHg | 19 | 86.6 (58.6–109.4) | 0.064 (-0.188–0.316) |
| 150≤SBP, mmHg | 23 | 74.9 (49.9–131.2) | 0.058 (-0.197–0.314) |
| DBP model |  |  |  |
| Subjects with insulin sensitivity in 10th-90th percentile^*^ | | |  |
| 60≤DBP<80, mmHg | 104 | 88.6 (66.7–127.8) | Ref. |
| 80≤DBP<90, mmHg | 70 | 92.1 (62.6–136.5) | 0.023 (-0.109–0.156) |
| 90≤DBP, mmHg | 41 | 113.0 (79.7–143.7) | 0.069 (-0.094–0.233) |
| Subjects without taking oral antihypertensive agents^†^ | | |  |
| 60≤DBP<80, mmHg | 113 | 91.7 (66.9–131.2) | Ref. |
| 80≤DBP<90, mmHg | 73 | 104.3 (64.6–145.2) | 0.113 (-0.019–0.245) |
| 90≤DBP, mmHg | 33 | 119.3 (85.2–171.0) | 0.132 (-0.045–0.310) |
| Subjects without accepting insulin treatment^‡^ | | |  |
| 60≤DBP<80, mmHg | 114 | 99.2 (70.0–136.2) | Ref. |
| 80≤DBP<90, mmHg | 74 | 101.9 (70.4–146.2) | 0.074 (-0.057–0.204) |
| 90≤DBP, mmHg | 36 | 123.0 (94.9–172.2) | 0.063 (-0.107–0.234) |
| Subjects with the duration of diabetes <1 year^§^ | | |  |
| 60≤DBP<80, mmHg | 65 | 101.4 (72.0–149.5) | Ref. |
| 80≤DBP<90, mmHg | 47 | 108.8 (78.2–155.7) | 0.105 (-0.081–0.291) |
| 90≤DBP, mmHg | 18 | 119.1 (77.8–173.3) | 0.067 (-0.214–0.348) |
| Subjects with the duration of diabetes ≥1 year^§^ | | |  |
| 60≤DBP<80, mmHg | 69 | 82.3 (60.7–127.6) | Ref. |
| 80≤DBP<90, mmHg | 41 | 74.9 (54.8–134.8) | 0.069 (-0.093–0.232) |
| 90≤DBP, mmHg | 31 | 112.1 (81.2–145.2) | 0.106 (-0.078–0.290) |
| ^*^Adjusted age, sex, duration of diabetes, BMI, log TG, LDL-C, log Matsuda index, insulin treatment, oral hypoglycemic agent treatment, ACE inhibitor/ARB treatment, and other antihypertensive agent treatment. ^†^Adjusted the same covariates in ^*^ except for ACE inhibitor/ARB treatment and other antihypertensive agent treatment. ^‡^Adjusted the same covariates in ^*^ except for insulin treatment. ^§^Adjusted the same covariates in ^*^ except for the duration of diabetes. Data of ISSI2 were median (25th– 75th). *b*(95%CI) was from linear regression analysis. ISSI2 was analyzed in models with the form of log transformation. ISSI2, insulin secretion-sensitivity index-2; SBP, systolic blood pressure; DBP, diastolic blood pressure; HbA1c, glycosylated hemoglobin; BMI, body mass index; TG, triglyceride; LDL-C, low-density lipoprotein cholesterol; ACE, angiotensin converting enzyme; ARB, angiotensin receptor blocker. | | | |
